# The Private Houses of London

**Published:** 1920-01-17

**Authors:** 


### ATMOSPHERIC POLLUTION.

### The Private Houses of London.

THE subject of atmospheric pollution is one of perennial interest, and one which has only very recently come to its own. Evelyn in his diary animadverts on the smoke of London, and compares its fog unfavourably with the mists of the country; and since then most writers on London have referred to these fogs. This attitude so typical of the *laissez-faire* of the time first underwent a change when the Public Health Act of 1875 became law. This Act makes it a "nuisance" for any chimney other than that of a private house to send forth black smoke, unless this is proved to be unavoidable, the nature of the work done precluding the use of a grate and furnace which will lead to the combustion of all the solid particles. Since then the Alkali, &c., Works' Regulation Act of 1906 regulates the discharge of acid gases into the air.

### RESULTS OF PAST MEASURES.

These have done much, and economy has done more, for it now has come about that the manufacturer can not afford to allow by-products to escape into the air, and so be lost; all such gases are collected by suitable scrubbers, and are converted into valuable material while in the matter of smoke, the more efficient the furnace, the less the waste of fuel as smoke. These factors have certainly contributed much to the freeing of the atmosphere from pollution, the pity is that the records of the actual degree of pollution in former time are wanting. Most old Londoners are agreed that the modern "London particular" will not bear comparison with those of fifty or even thirty years ago, but we have no records giving the weight of suspended solids then in the air, nor their composition.

### THE PRESENT PROBLEM.

The problem at present almost untackled is that of the private house. There is probably no method of heating at once so uneconomical, so inefficient, and yet so pleasant and popular as the old-fashioned coal-fire—in its disparagement almost anything can be truthfully said—nothing in its favour except that it is a pleasanter and more companionable form of warmth than any other; next in order of inefficiency and comfort comes the gas-stove, the other means of domestic heating are nowhere, and it is to these two, the open fire and the gas-stove, that a great part of the present pollution are due.

The incomplete combustion of the coal-fire provides a large quantity of tarry material, carbon as soot, sulphates, sulphides, and ammonia; the gas-fire yields ammonia and unsaturated carbon compounds; and in these days of bad gas, sulphates and sulphides, in comparatively large amounts. However, it is the open coal-fire which is the great offender, and must account for by far the greatest measure of pollution.

The report of the Advisory Committee on Atmospheric Pollution, published in *The Lancet* of November 15, is in this connection of the greatest interest: it gives, for the year 1918-19, all the figures which are needed to survey the subject, all those figures which we would like to have for the last fifty years at least. That they are only available for the last five reflects no discredit on the energetic body who are now producing them. From this report one learns that the weight of suspended solids in the air varies from a minimum of forty-eight metric tons per cubic kilometer per annum in the case of an observation station on Wandsworth Common to 266 tons at Embankment Gardens. For comparison, the weight of rain falling on the same area over the same period when the rainfall is 586 mm. is 586,000 metric tons. That the contamination of the atmosphere should approach 0.5 per cent. of the rainfall is certainly a state of affairs which it should be possible to remedy, and only by the study of what is in the air can means be found to prevent the contamination.

### SOLID MATTER IN AIR.

The solid matter collected from the air is divided into two broad classes—the soluble in water and that insoluble—and of these the insoluble matter is subdivided into tar, other carbonaceous matter and ash, while the soluble is divided into ash, and that other part which is lost on ignition, in it also the sulphates, chlorides and ammonia are estimated.

Glancing at the curves which illustrate the variations of these different constituents for 1918-1919, there seems to be a very obvious relationship between many of them and the rainfall, though the maximum rainfall is in September while solids are greatest in October. This is unexpected, as one would have expected the rain to bring down the suspended matter. Tar, insoluble carbonaceous material, ammonia, and to a less extent chlorine remain almost constant, the other components varying between very wide limits. These curves do not agree very well with those based on the combined figures of 1915-1919, and this brings out still more the need of a prolonged series of experiments especially as these last years have been so complicated by the springing up of factories and the shortage and rationing of coal. Another much needed advance will be made when readings are obtained from a greatly increased number of sources. At present Malvern is the only country area where estimations are made, and it certainly seems a pity that those large health resorts where so much attention is paid to the meteorology, do not turn their attention to this subject. Furthermore, if a few stations at places remote from towns could be inaugurated they would be of the utmost value in determining to what extent the comparatively pure air of the country was polluted.

